# Risk assessment of peanut protein traces found in refined peanut oil

**DOI:** 10.1186/2045-7022-5-S3-O13

**Published:** 2015-03-30

**Authors:** Marty Blom, Astrid Kruizinga, Rene Crevel, Geert Houben

**Affiliations:** 1 TNO, Zeist, The Netherlands; 2 Unilever, Bedford, UK

## 

Highly refined peanut oil is considered to pose a risk to people with peanut allergy.[1] Although that risk has not been characterised, but controlled clinical challenges suggest it is negligible. A consequence is that the risk from cross contact between other refined vegetable oils and refined peanut oil during production must be assessed in order to assure consumer safety. Use of these refined vegetable oils in consumer food products potentially leads to very small amounts of peanut proteins in the final product. However, it is not clear yet if these amounts could reach levels that are relevant for public health. Allergen risk assessment using probabilistic techniques enables quantitative estimation of the risk after the consumption of a product that unintendedly contains an allergen.

Several scenarios were defined for the unintended allergen levels in food products based on the estimated production scale of refined peanut oil in the UK, estimated figures for cross-contact and the peanut protein concentration in refined peanut oil. The consumption of 12 food products, selected for their high contribution to fat consumption including biscuits, margarine, ice cream and fried food, was combined with the peanut dose distribution. Food consumption data were derived from the National Diet and Nutrition Survey in the UK, and the Dutch National Food Consumption Survey. Peanut dosedistribution data were the same as used for the scientific review of VITAL^®^ (Voluntary Incidental Trace Allergen Labelling).[2] We estimated that the amounts and distributions of peanut proteins posed a risk of objective allergic reactions in 0.03 to 0.6% of the peanut-allergic consumer population. The concentrations of peanut protein in selected food products would be below the limit of detection of current analytical methods and in all the scenarios examined, which included worst-case, the amounts consumed were below the VITAL reference dose for peanut. On that basis, the health risk is very small and none of those products would warrant precautionary labelling.
